# Efficient Generation of Induced Pluripotent Stem Cell-Derived Definitive Endoderm Cells with Growth Factors and Small Molecules

**DOI:** 10.3390/cells14110815

**Published:** 2025-05-30

**Authors:** Faizal Z. Asumda, Shadia Alzoubi, Kiyasha Padarath, Nina John, Kimya Jones, Ravindra Kolhe, Ashis Kumar Mondal, Tae Jin Lee, Wenbo Zhi, Robert C. Huebert, Nathan P. Staff, Lindsey A. Kirkeby

**Affiliations:** 1 Department of Pediatrics, Medical College of Georgia, Augusta University, Augusta, GA 30912, USA; salzoubi@augusta.edu (S.A.); kpadarath@augusta.edu (K.P.); 2 Department of Pathology, Medical College of Georgia, Augusta University, Augusta, GA 30912, USA; kjones2@augusta.edu (K.J.); rkolhe@augusta.edu (R.K.); amondal@augusta.edu (A.K.M.); 3 Medical College of Georgia, Augusta University, Augusta, GA 30912, USA; nijohn@augusta.edu; 4 Center for Biotechnology and Genomics, Medical College of Georgia, Augusta University, Augusta, GA 30912, USA; talee@augusta.edu; 5 Vascular Biology Center, Medical College of Georgia, Augusta University, Augusta, GA 30912, USA; wzhi@augusta.edu; 6 Division of Gastroenterology and Hepatology, Mayo Clinic, Rochester, MN 55905, USA; huebert.robert@mayo.edu; 7 Department of Neurology, Mayo Clinic, Rochester, MN 55905, USA; staff.nathan@mayo.edu; 8 Center for Regenerative Biotherapeutics, Mayo Clinic, Rochester, MN 55905, USA; kirkeby.lindsey@mayo.edu

**Keywords:** definitive endoderm, induced pluripotent stem cell cells, small molecule, growth factor

## Abstract

Definitive endoderm (DE) differentiation leads to the development of the major internal organs including the liver, intestines, pancreas, gall bladder, prostate, bladder, thyroid, and lungs. The two primary methods utilized for *in vitro* differentiation of induced pluripotent stem cells (iPSCs) into DE cells are the growth factor (GF) and the small molecule (SM) approaches. The GSK-3 inhibitor (CHIR99021) is a key factor for the SM approach. Activin A and Wnt3a are utilized in the GF approach. In this study, both the GF and SM protocols were compared to each other. The results show that both the GF and SM protocol produce DE with a similar morphological phenotype, gene and protein expression, and a similar level of homogeneity and functionality. However, on both the gene expression and proteomic level, there is a divergence between the two protocols during hepatic specification. Proteomic analysis shows that hepatoblasts from the GF protocol have significantly differentially expressed proteins (DEPs) involved in liver metabolic pathways compared to the SM protocol. Well-validated DE differentiation protocols are needed to fully unlock the clinical potential of iPSCs. In the first step of generating DE-derived tissue, either protocol can be utilized. However, for hepatic specification, the GF protocol is more effective.

## 1. Introduction

**Figure 1:**
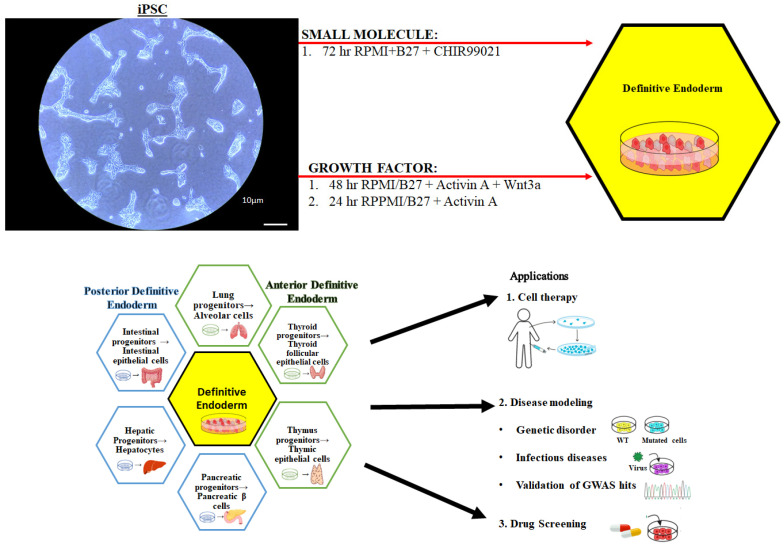
**The differentiation of definitive endoderm and its applications.** The definitive endoderm is represented in the black hexagon in the center image. Differentiation of the endoderm is separated into the anterior (green) and the posterior (blue) progenitors. On the right is the three major applications of definitive endoderm and its progenitors: (1) cell therapy, (2) disease modeling, and (3) drug screening.

Organogenesis in the developing embryo is a complex developmental process that depends on cell signaling, proliferation, and differentiation [1]. Early embryonic gastrulation results in the development of three germ cells (ectoderm, mesoderm, and endoderm) [1,2]. Definitive endoderm (DE) differentiation and patterning gives rise to the major internal organs including the liver, intestines, pancreas, gall bladder, prostate, bladder, thyroid, and lungs (Figure 1) [1,2]. These endoderm-derived organs vary significantly in their form and function (absorption, gas exchange, detoxification, metabolism, or glucose homeostasis) [1]. Endoderm cells are difficult to access in the *in utero* embryo and are a significantly small cell population within the embryo [3]. The generation and culture of embryonic stem cells (ESCs) and induced pluripotent stem cells (iPSCs) has enabled the in vitro differentiation of endoderm-derived tissue and organ-specific cells [4,5,6,7]. These stem cell models (Figure 1) enable the in vitro characterization of endoderm morphogenesis and cell behavior.

The differentiation of human iPSC lines into specific organ or tissue cell models is hampered by cell line and clonal variability. The differentiation potential of each cell line and each iPSC clone from the same line is often not uniform, with variable degrees of homogeneity in the final differentiated cell population. There are multiple factors that may account for the inter-line variability. This includes the culture conditions versus the process of reprogramming and the resulting epigenetic effects. Derivation of a homogeneous population during DE differentiation is especially critical given the multi-step approach that is required to generate endoderm-derived organs and tissues [6,7]. Well-validated DE differentiation protocols are needed to fully unlock the clinical potential of induced pluripotent stem cells. The two primary methods utilized for in vitro differentiation of iPSCs into DE cells are the growth factor (GF) and the small molecule (SM) approaches. In vertebrates, the transforming growth factor-β (TGFβ) ligand Activin A and NODAL, fibroblast growth factor (FGF), and WNT growth factor pathways are required for DE specification [1,4,5,6,7]. Similarly, in vitro GF protocols for endoderm specification have utilized Activin A/NODAL, Wnt3a/β-Catenin, bone morphogenic protein (BMP), and bFGF [5,6,7,8,9,10,11,12]. SMs, including glycogen synthase 3 (GSK3) inhibitors (CHIR99021 or BIO) and phosphatidyl-inosotol-3-kinase (PI3K) inhibitors, have been proposed as a simple and more cost-effective approach for the generation of iPSC-derived DE cells [13,14,15,16,17,18]. The GSK-3 inhibitor (CHIR99021) which modulates NODAL gene expression and activates Wnt-signaling can be used as a single agent in culture media to effectively generate homogeneous DE cells [17,18,19,20]. Our group has been focused on the development of patient-specific in vitro models of hepatocytes, pancreatic cells, and cholangiocytes for the purposes of in vitro viral infection, metabolomics, drug screening and toxicology, and as potential model systems for carcinogenesis. Our aim in these studies was to test the hypothesis that both growth factors and small molecules produce a homogenous definitive endoderm population in the first step of iPSC differentiation, but subsequent steps produce more heterogenous populations of specific organ cell types.

## 2. Materials and Methods

### 2.1. Small Molecules, Chemicals, Growth Factors, and Antibodies

Hepatocyte growth factor (HGF) (294HGN100), recombinant Wnt-3a (5036-WN), and Activin A (338-AC) were from R&D Systems, Minneapolis, MN, USA. Dimethyl sulfoxide (DMSO) (D5879) was from Sigma Aldrich (St. Louis, MO, USA). Stemolecule CHIR99021 (04–0004) was from Stemgent (Lexington, MA, USA). 2-Mercaptoethanol (M6250), RPMI medium (61870036), B27 supplement (17504044), Glutamax supplement (35050061), Insulin–Transferrin–Selenium supplement (41400045), DMEM (10829018), Knockout serum replacement (10828028), non-essential amino acids solution (11140050), Dulbecco’s phosphate-buffered saline, calcium and magnesium free DPBS^−/−^ (14190144), ProLong Gold Antifade Mountant with DAPI (P36935), and Trizol Reagent (15596026) were from Thermo Fisher Scientific (Rockford, IL, USA). Antibodies against SOX17 (sc-130295), FOXA2 (sc-271103), CXCR4 (sc-53534), HNF4A (sc-374229), and AFP (sc-80464) were from Santa Cruz Biotechnology, Inc. (Santa Cruz, CA, USA). Secondary FITC-conjugated anti-rabbit (A21206) and anti-mouse (A11059), and TRITC-conjugated anti-rabbit (R37117) and anti-mouse (A11005) antibodies were from Thermo Fisher Scientific (Rockford, IL, USA). iScript cDNA Synthesis Kit (1708891) and iTAQ Universal SYBER Green Supermix (1725124) were from Bio-Rad (Hercules, CA, USA).

### 2.2. Human Pluripotent Stem Cells

A total of 15 healthy human iPSCs were sourced from the Mayo Clinic Biotrust (Rochester, MN, USA) and utilized for these experiments. Human iPSCs were cultured as previously described [21].

### 2.3. Small Molecule Definitive Endoderm Protocol

Human iPSCs cultured in 6-well dishes to 60% confluence were washed with RPMI/B27 (Thermo Fisher Scientific, Rockford, IL, USA) and cultured in RPMI/B27/Glutamax/penicillin/streptomycin with Insulin–Transferrin–Selenium supplement and 6uM CHIR99021 (Stemgent, Lexington, MA, USA) for 72 h at 37 °C, 5% CO_2_ with daily media changes. CHIR99021 was removed after 72 h and DE cells were cultured in RPMI/B27/Glutamax/penicillin/streptomycin + Insulin–Transferrin–Selenium supplement for 24 h (Figure 1) [19,20,21].

### 2.4. Growth Factor Definitive Endoderm Protocol

Human iPSCs cultured in 6-well dishes to 60% confluence were washed with RPMI/B27 (Thermo Fisher Scientific, Rockford, IL, USA) and cultured in RPMI/B27/Glutamax/penicillin/streptomycin with Insulin–Transferrin–Selenium supplement, Activin A (100 ng/mL), and Wnt3a (25 ng/mL), for 48 h, and with Activin A (100 ng/mL) for another 24 h at 37 °C, 5% CO_2_ with daily media changes (Figure 1).

### 2.5. Small Molecule Hepatoblast Specification Protocol

DE cells were washed with DPBS^−/−^ and DMEM medium (Thermo Fischer Scientific, Rockford, IL, USA) and cultured in hepatoblast medium for 6 days. SM hepatoblast specification medium contained DMEM (Thermo Fisher Scientific, Rockford, IL, USA), 10% Knockout serum replacement, 1% DMSO (Sigma, St. Louis, MO, USA), 5mM Glutamax (Thermo Fisher Scientific, Rockford, IL, USA), 1% non-essential amino acids (Thermo Fisher Scientific, Rockford, IL, USA), 1% penicillin/streptomycin, and 100 µM 2-mercaptoethanol (Sigma, St. Louis, MO, USA) [19,20,21].

### 2.6. Growth Factor Hepatoblast Specification Protocol

DE cells were washed with DPBS^−/−^ and DMEM/F12 and cultured in hepatoblast specification medium for 6 days. GF hepatoblast specification medium contained DMEM/F12, 10% KOSR, 1% Glutamine, 1% non-essential amino acids, and 1% penicillin/streptomycin containing 100 ng/mL of hepatocyte growth factor (HGF) and 1% dimethyl sulfoxide (DMSO) [22].

### 2.7. Immunocytochemistry

Immunocytochemistry staining was completed as previously described [21]. Confocal images were obtained using 60 × oil objective on a Leica SP5 Scanning Confocal Microscope (Frankfurt, Germany). Image J (National Institutes of Health and the Laboratory for Optical and Computational Instrumentation (LOCI, University of Wisconsin) and Adobe Photoshop (Adobe Systems, San Jose, CA, USA) were used for processing and analysis. Images were randomized, and a minimum of 300 positively stained differentiated cells were counted in 4 separate fields of view for quantification.

### 2.8. RNA Isolation, cDNA Synthesis, and RT-PCR Analysis

Total RNA was extracted, and qPCR was completed as previously described [21]. Gene analysis was performed with the Bio-Rad CFX Manager software Version 3.1 (Bio-Rad, Hercules, CA, USA). Gene expression was normalized relative to unstimulated cells and fold variation was GAPDH-normalized. The primer sequences used are shown in Table 1.

### 2.9. Proteomics, Metabolomics, and LC-MS/MS Analysis

Proteomics and metabolomics analyses were conducted through the Medical College of Georgia Cancer Center Proteomics and Metabolomics Core. Detailed methods for protein extraction, precipitation, reduction, alkylation, digestion, and LC-MS/MS analysis are published by Glass et al. [23].

#### 2.9.1. Bioinformatics Analysis

Volcano plots and heat maps were generated using https://www.bioinformatics.com.cn/, accessed on 12 November 2024.

#### 2.9.2. Pathway Analysis

A list of significantly differentially expressed proteins (*p*-value ≤ 0.05) was analyzed using ShinyGO version 0.741: a graphical gene-set enrichment tool for animals and plants (http://bioinformatics.sdstate.edu/go, accessed on 30 December 2024).

#### 2.9.3. STRING Analysis

The STRING (Search Tool for the Retrieval of Interacting Genes/Proteins) database was used for the illustration of predicted interactions of identified proteins and neighbor genes. The proteins significantly differentially expressed in the different groups were processed in STRING version 12.0 (https://string-db.org/, accessed on 30 December 2024) to obtain medium-confidence interaction data (score ≥ 0.7). The PPI network was visualized using the Cytoscape 3.2.1 software (https://cytoscape.org/, accessed on 30 December 2024).

#### 2.9.4. Imaging

Phase contrast imaging was performed on the EVOS Cell Imaging System (Thermo Fisher Scientific, Rockford, IL, USA).

#### 2.9.5. Statistical Analysis

All differentiation experiments were carried out in triplicate (n = 3). Data are presented as mean ± SEM. Statistical significance was determined using two tailed Student’s *t*-test with *p* < 0.05 determined to be significant.

## 3. Results

### 3.1. Characterization of Definitive Endoderm Cells

**Figure 2:**
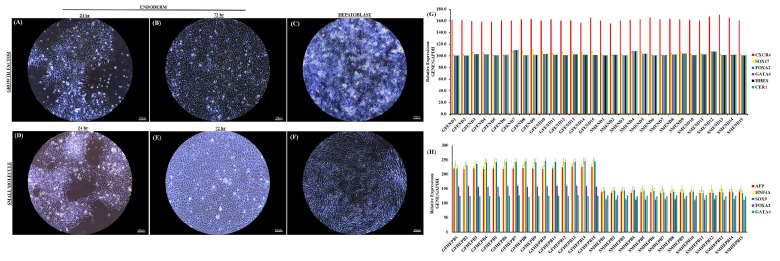
**Gene expression of endoderm and hepatoblast cells.** Representative phase contrast micrographs of growth factor endoderm cells (GF-ENDs) (**A**,**B**), growth factor hepatoblast cells (GF-HEPBs) (**C**), small molecule endoderm cells (SM-ENDs) (**D**,**E**), and small molecule hepatoblast cells (SM-HEPBs) (**F**), showing morphological changes after differentiation (20×). qRT-PCR analysis for relative expression of endoderm genes in GF-END and SM-END cells (**G**), and hepatoblast genes in GF-HEPB and SM-HEPB cells (**H**). Columns show the combined mean ΔΔC_t_ values for each marker. Data represent relative expressions of transcripts normalized relative to GAPDH and undifferentiated controls. Data are represented as mean ± SEM for three biologically independent experiments (n = 3).

The small molecule endoderm (SM endoderm) and growth factor endoderm (GF endoderm) follow a similar pattern of differentiation. In the first 24 h of exposure to specification medium, we noted a change from the typical flat monolayer iPSC colony morphology to a domed appearance with bright 3D structures at the center of each iPSC colony. In both the SM and GF conditions, significant endoderm cell migration and proliferation out of the colonies is observed, with a decreased nuclear to cytoplasmic ratio within the first 24 h (Figure 2A,D and Supplement Figure S1). By 72 h, a well-defined monolayer cell population with distinct single-cell borders and a cobblestone/petal appearance is noted for both culture conditions (Figure 2B,E). At 72 h, a well-defined homogenous population of DE cells is noted for both culture conditions with no evidence of iPSCs. The morphological appearance of DE cells at the end of 72 h is uniform across all 15 iPSC lines for both SM and GF culture conditions (Supplemental Figure S1). The key to deriving a uniform homogenous definitive endoderm population based on these experiments is to start differentiation at no greater than 60% confluency. At higher (>80%) confluence, the iPSC colonies fail to completely undergo the full process of differentiation.

**Figure 3:**
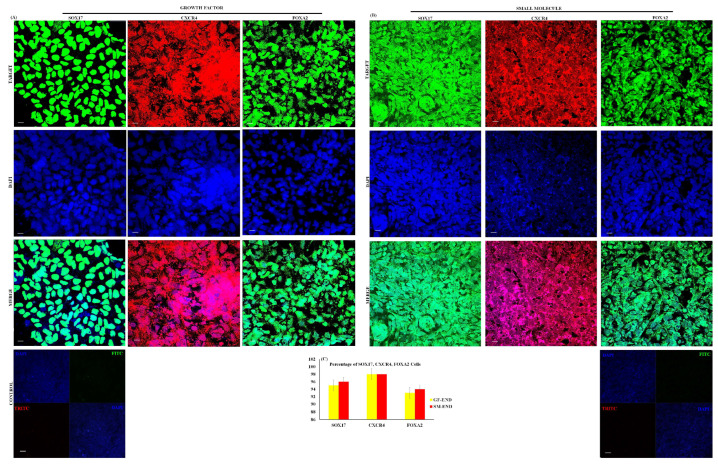
**Protein expression of endoderm cells.** Immunostaining of endoderm protein expression in growth factor endoderm cells (GF-ENDs) (**A**) and small molecule endoderm cells (SM-ENDs) (**B**). Percentage expression of proteins (**C**). Nuclei were stained with DAPI in all. Representative data from three independent experiments are shown.

Gene and protein expression analysis at the end of 72 h of differentiation demonstrates a uniform level of expression of DE markers (Figure 2G). CXCR4, SOX17, FOXA2, CER, GATA4, and HHEX gene expressions are significant and uniform across all 15 iPSC lines under both SM and GF culture conditions (Figure 2G). The results of qPCR analysis show that CXCR4 gene expression is the highest of the DE markers across all 15 cell lines under both culture conditions (Figure 2G). Immunostaining of the three key DE markers CXCR4 (98%), SOX17 (96%), and FOXA2 (96%) demonstrate high differentiation efficiency and homogeneity for both SM and GF conditions across all 15 cell lines (Figure 3A–C).

**Figure 4:**
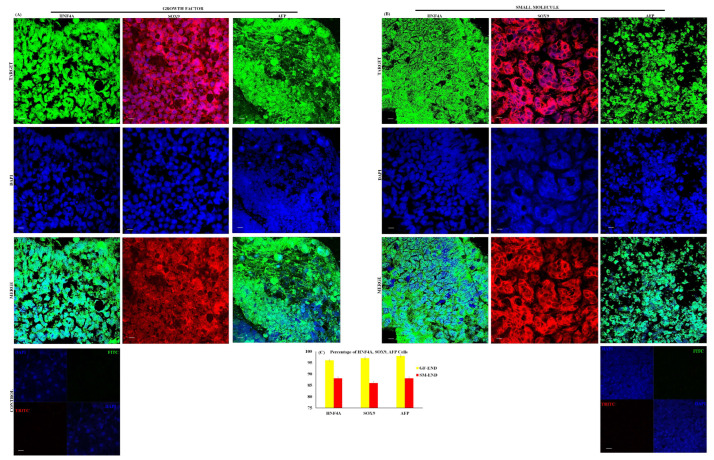
**Protein expression of hepatoblast cells.** Immunostaining of hepatoblast protein expression in growth factor hepatoblast cells (GF-HEPBs) (**A**) and small molecule hepatoblast cells (SM-HEPBs) (**B**). Percentage expression of proteins (**C**). Nuclei were stained with DAPI in all. Representative data from three independent experiments are shown.

We tested the downstream specification potential of SM- and GF-derived DE cells by generating hepatoblast cells. GF and SM hepatoblast cells are distinct in morphology (Figure 2C,F). Individual GF hepatoblast cells are larger and take on a progressive cuboidal shape, with well-defined borders and with significant lipid production and cytoplasmic granularity (Figure 2C and Supplemental Figure S1). SM hepatoblast cells have a mesenchymal ball-and-stick-like appearance with less lipid production (Figure 2F and Supplemental Figure S1). The expression of AFP, HNF4A, SOX9, FOXA2, and GATA4 by qPCR is significantly higher in hepatoblast cells derived with the GF approach (Figure 2H). AFP, HNF4A, and SOX9 have the highest levels of expression across all 15 cell lines for GF- and SM-derived hepatoblast cells (Figure 2H). Evaluation of AFP, HNF4A, and SOX9 protein expression by immunocytochemistry is shown in Figure 4A,B. All three hepatoblast proteins are uniformly expressed at sufficiently high levels, although the GF hepatoblast cells have a greater percentage of positive cells (Figure 4C).

### 3.2. Proteomics of Endoderm and Hepatoblast Cells

**Figure 5:**
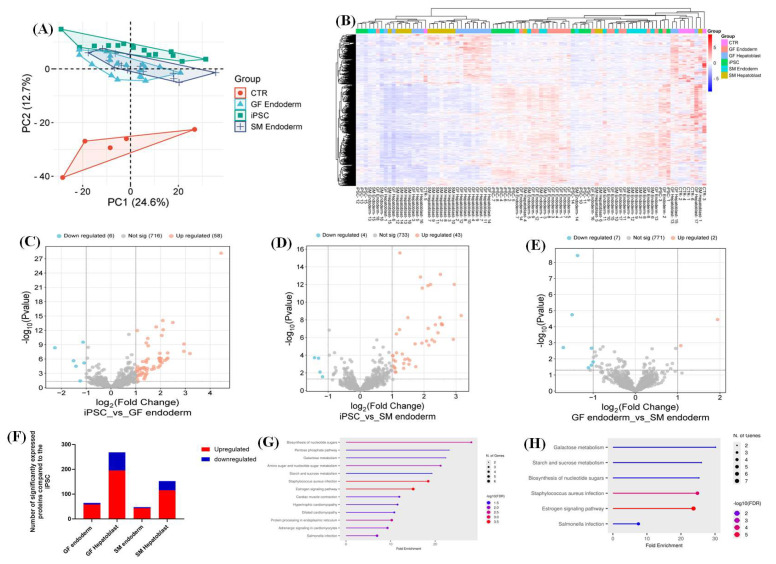
**Comparison of GF endoderm and SM endoderm.** Principal component analysis (PCA) showing the various cell types: CTR, iPSCs, GF endoderm, and SM endoderm as different shapes (**A**). Clustering is depicted for the individual groups with similar characteristics. The CTR is represented in red, iPSC in green, GF endoderm in light blue, and SM endoderm in dark blue. The PCA plot was generated using peptide abundance data of all peptides analyzed per cell type, with 10 replicates. A comparative heatmap of all the replicates per cell type (iPSC, CTR, GF endoderm, and SM endoderm) and the identified protein groups (**B**). Volcano plots of the differentially expressed proteins from the different cell types, GF and SM endoderm, against the iPSC were generated (**C**–**E**). The negative x-axis represents downregulation (blue) in the cell type compared to the control, and the positive axis represents upregulated (red) proteins in the different cell types compared to the control. A bar graph showing the number of significantly upregulated (red) and downregulated (blue) proteins in each cell type compared to the iPSCs (**F**). Proteomic pathway analysis of significant differentially expressed proteins compared to CTR (**G**,**H**). A dot plot was generated using the uniquely differentially expressed proteins in ShinyGO analysis, with KEGG pathway enrichment and fold enrichment based on the number of genes present in each pathway. The FDR cut-off was set at 0.05, and the number of pathways was set to 20 (**G**,**H**).

The proteomes of iPSCs, SM endoderm and hepatoblasts (SM endoderm and SM hepatoblasts), GF endoderm and hepatoblasts (GF endoderm and GF hepatoblasts) and controls (CTR) (HUH7, HEP3B, HEPG2, SNU398, and PHHs) were analyzed by data-dependent acquisition mass spectrometry (DDA-MS). Principal component analysis (PCA) showed an overlap between GF endoderm, SM endoderm, and the iPSCs (Figure 5A). The heatmap showed a global change in upregulated and downregulated proteins from the undifferentiated state (iPSC) to the differentiated state (GF endoderm, SM endoderm, SM hepatoblast, GF hepatoblast, and CTR) (Figure 5B). Volcano plots were generated to identify significant and differentially expressed proteins in the distinct groups (Figure 5C–E).

Significant differentially expressed proteins were classified as upregulated versus downregulated. A minimum fold change ≥ 1.5, a false discovery rate (FDR) of 1%, and an adjusted *p*-value (q-value) ≤ 0.05 were used to filter proteins that were significantly different between the iPSC, GF endoderm, and SM endoderm (Figure 5C–E). The GF endoderm (64 DEPs) had a slightly higher number of significantly differentiated proteins (DEPs) in relation to the SM endoderm (47 DEPs) when compared to the iPSCs (Figure 5F). To understand the variation in DEPs between groups, we categorized the DEPs with ShinyGO analysis based on the specific biological pathways they affect. The GF endoderm had unique signaling pathways that were not present in the SM endoderm when compared to iPSCs (Figure 5G,H). These pathways included the following: amino sugar and nucleotide sugars metabolism, pentose phosphate pathways, protein processing in the endoplasmic reticulum, and cardiac metabolism pathways. Common pathways between the GF and SM endoderm included the following: galactose metabolism, starch and sucrose metabolism, biosynthesis of nucleotide sugar, and estrogen signaling pathway (Figure 5G,H). We conducted a direct comparison of the proteome of the GF endoderm and SM endoderm datasets. The endoderm derived from the GF and SM protocols have a 98.97% similarity in the total proteins between the two datasets. Suggesting that there is no difference between DE cells derived from SM and GF.

**Figure 6:**
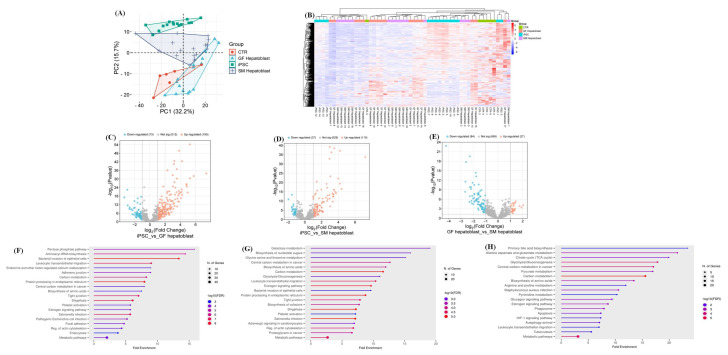
**Comparison of GF endoderm and SM endoderm.** Principal component analysis (PCA) showing the various cell types as different shapes: CTR, iPSC, GF hepatoblast, and SM hepatoblast (**A**). Clustering is depicted for the individual groups with similar characteristics. The CTR is represented in red, iPSC in green, GF hepatoblasts in light blue, and SM hepatoblasts in dark blue. The PCA plot was generated using peptide abundance data of all peptides analyzed per cell type, with 10 replicates. Comparative heatmap of all the replicates per cell type (iPSC, CTR, GF hepatoblasts, and SM hepatoblasts) and the identified protein groups (**B**). Volcano plots of the differentially expressed proteins from the different cell types, GF and SM hepatoblasts, against the iPSC were generated (**C**–**E**). The negative x-axis represents downregulation (blue) in the cell type compared to the control, and the positive axis represents upregulated (red) proteins in the different cell types compared to the control. Proteomic pathways analysis of significant differentially expressed proteins compared to CTR (**F**–**H**). A dot plot was generated using the uniquely differentially expressed protein in ShinyGO analysis, with KEGG pathway enrichment and fold enrichment based on the number of genes present in each pathway. The FDR cut-off was set at 0.05, and the number of pathways was set to 20 (**F**–**H**).

We analyzed the hepatoblasts from both protocols. The PCA and heatmap showed that the GF hepatoblast, SM hepatoblast, and CTR groups had a wider distribution than the iPSCs (Figure 6A,B). The GF hepatoblast was the only group that overlapped with the control group (HUH7, HEP3B, HEPG2, SNU398, and PHHs) (Figure 6A). Volcano plots were generated to identify significant and differentially expressed proteins in the distinct groups, using the parameters previously described (Figure 6C–E). The DEPs were then classified into their corresponding pathways. Compared to the iPSCs, the GF hepatoblast and SM hepatoblast groups had 11 pathways in common (Figure 6F,G). These pathways included the following: regulation of actin cytoskeleton, metabolic pathways, estrogen signaling pathway, platelet activation, tight junction, biosynthesis of amino acids, protein processing in the endoplasmic reticulum, carbon metabolism, and leukocyte transendothelial migration. The GF hepatoblasts showed the highest number of DEPs compared to the iPSCs, which suggests a higher level of differentiation efficiency in comparison to the SM hepatoblasts (Figure 5F).

**Figure 7:**
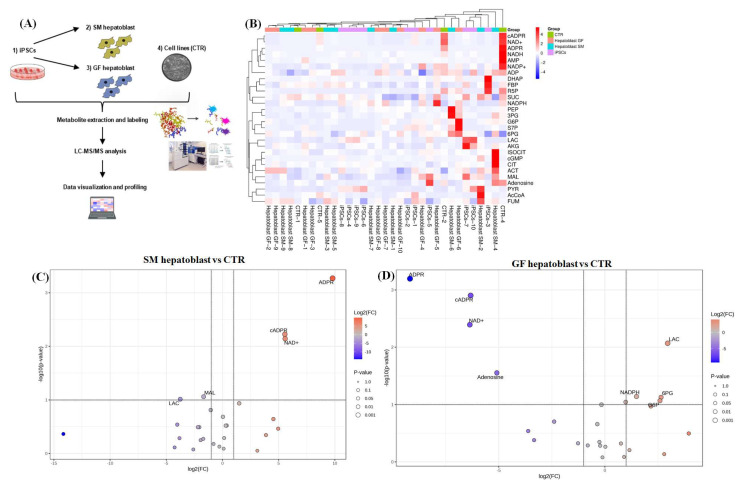
**Comparison of TCA metabolites of both SM and GF hepatoblasts compared to the control.** Diagrammatic representation of the targeted TCA cycle LC-MS workflow (**A**). A comparative heatmap of the metabolic profile of 15 iPSCs, SM hepatoblast, GF hepatoblast, and the CTR group replicated to the labeled metabolites (**B**). Volcano plots of the differentially expressed metabolites from the SM hepatoblast against the CTR group (**C**). The negative x-axis represents downregulated (blue) metabolites compared to the CTR, and the positive axis represents upregulated (red) metabolites compared to the control. Volcano plots of the differentially expressed metabolites from the GF hepatoblast against the CTR group (**D**). The negative x-axis represents downregulated (blue) metabolites compared to the CTR, and the positive axis represents upregulated (red) metabolites compared to the control.

The direct comparison between the SM hepatoblast and GF hepatoblast groups showed an 85.7% similarity between the proteomes of the two datasets. This is a 13.2% drop in similarity compared to the direct comparison of the DE cells from the two protocols. Proteomic analysis showed that the significantly differentially expressed proteins are involved primarily in metabolic pathways, including carbon metabolism, amino acid biosynthesis, pyruvate metabolism, glucagon signaling, estrogen signaling, pentose phosphate pathway, HIF-1 signaling, TCA cycle, and glycolysis/gluconeogenesis (Figure 6H). This suggests that a primary difference between the GF and SM protocols in terms of their individual effect on iPSCs during differentiation is the extent of metabolic rewiring and maturation of the endoderm into hepatoblasts. To investigate this hypothesis, metabolites of the TCA cycle were compared across the datasets using a targeted LC/MS approach (Figure 7A,B). The TCA metabolites from SM endoderm and GF endoderm showed slight variation, with five significantly differentially expressed TCA metabolites (S7P, PYR, ADPR, NADPH, and NADH) out of a panel of 28 metabolites tested (Supplementary Figure S2). The SM hepatoblasts and GF hepatoblasts exhibited six significantly differentially expressed TCA metabolites (PYR, NADPH, cGMP, ISOCIT, R5P, and DHAP) out of the same panel of 28 metabolites (Supplementary Figure S2). Comparison of hepatoblasts from the two different protocols to the CTR group showed significant differences in metabolite expression (Figure 7C,D). GF hepatoblasts showed significantly increased expression of 6PG, GP6, NADPH, and LAC, along with significantly decreased expression of ADPR, cADPR, NAD+, and adenosine (Figure 7C). In contrast, SM hepatoblasts displayed significantly increased expression of ADPR, cADPR, and NAD+, while LAC and MAL were significantly decreased (Figure 7D). These findings suggest that the TCA cycle metabolites differ between the two differentiation protocols and show distinct metabolic profiles when compared to the CTR group, highlighting potential variations in metabolic regulation.

## 4. Discussion

We have presented morphological, gene and protein expression, and proteomics and metabolomic evidence that small molecules (SMs) and growth factors (GFs) have similar definitive endoderm (DE) cell differentiation efficiency. Both protocols produce DE cells with similar levels of homogeneity and functionality. We demonstrate that both SM- and GF-derived DE cells express similar levels of key DE markers (CXCR4, SOX17, FOXA2, CER, GATA4, and HHEX). The expression of CXCR4 is a distinguishing feature for committed endoderm progenitor cells, which arise from mesendoderm after 24 h of induction [9]. These CXCR4-positive committed endoderm progenitor cells further differentiate into more mature DE cells characterized by expression of SOX17, FOXA2, and HHEX [9]. In our experiments, CXCR4 gene and protein expression is uniformly the highest in both the SM- and GF-derived DE cells across all 15 cell lines (Figure 2G,H). The DE is a vital precursor for internal organs such as the liver and pancreas. Suboptimal DE induction is the likely cause of cell line-to-cell line and experiment-to-experiment variability. The non-DE cell types within the culture can contribute to the development of heterogeneous end-stage populations. By day 3 of differentiation, both the SM and GF protocols produce DE cells that are uniformly mature.

To test the functionality of these DE cells, we generated hepatoblast cells by utilizing well-established SM [20,21] and GF [22] hepatic specification protocols. Our hepatic specification experiments demonstrate that the specific factors utilized in these studies for SM (CHIR99021) and GF (Activin A and Wnt3a) differentiation of DE from iPSCs are equally effective. However, in the subsequent steps of differentiation, GFs are superior to SMs, as demonstrated by gene (Figure 2H) and protein (Figure 4) expression. Using a DDA MS-based quantitative proteomic approach, a comparative analysis of the whole proteome of iPSCs differentiated into endoderm and hepatoblast, with SMs and GFs, were compared to a control group consisting of hepatocyte-derived carcinoma cell lines and primary human hepatocytes. The different cell types (iPSCs, GF endoderm, SM endoderm, GF hepatoblast, SM hepatoblast, and CTR) were analyzed by principal component analysis (PCA), which showed that the global protein abundances in the GF endoderm and SM endoderm are similar. The PCA plots of GF endoderm and SM endoderm overlapped with each other (Figure 5A). When a direct comparison was made between GF and SM endoderm, no significant differences in enriched pathways were detected. An efficient protocol to differentiate iPSCs into endoderm is essential for regenerative medicine and for modeling diseases. In this study, no significant proteomic differences were observed between the endoderm from the SM and GF protocols. Therefore, both protocols can be used to effectively differentiate the iPSCs into definitive endoderm.

A divergence in proteomic profiles becomes apparent between the two protocols during hepatic specification. Hepatoblasts derived from the GF protocol exhibited a distinctly different proteome, with an increased number of differentially expressed proteins (DEPs) compared to those derived from the SM protocol (Figure 6). Notably, these differences are associated with metabolic pathways (Figure 6). Hepatocyte growth factor (HGF) is a key regulator in hepatic progenitor regeneration and maintenance. It influences glucose transport and metabolism [24]. In addition to its role in endoderm differentiation into hepatoblasts, HGF is essential for metabolically reprogramming cells to adopt a hepatocyte-like metabolism [24,25]. The metabolomic profiles of SM hepatoblasts and GF hepatoblasts differed significantly when compared to the CTR group. GF hepatoblasts exhibited a significant increase in TCA cycle metabolites primarily involved in glycolysis and gluconeogenesis, including 6PG, GP6, and NADPH (Figure 7D), which are characteristic of human hepatocytes [26,27,28]. In contrast, SM hepatoblasts showed a significant increase in TCA metabolites associated with both canonical and non-canonical energy metabolism pathways, such as ADPR, cADPR, and NAD+, which are predominantly linked to cancer-related metabolic reprogramming [29,30]. These findings suggest distinct metabolic adaptations between the two differentiation protocols, with GF hepatoblasts aligning more closely with normal hepatic metabolism, while SM hepatoblasts exhibit metabolic traits commonly observed in tumorigenic processes. Therefore, the observed variations between the two protocols may be attributed to differences in the way HGF and other metabolites influence cellular metabolism, ultimately impacting the maturation and functionality of the resulting hepatoblasts.

There are limitations to these studies, including the fact that our data are primarily observational. We have drawn conclusions based on the data presented, but additional mechanistic and functional studies are required. The reproducibility of the two differentiation protocols is a key question, and we have tried to address this issue by utilizing a total of 15 different iPSC lines to ensure that the observed differences are reflective of a true effect. These limitations notwithstanding, the data presented here were generated by repeating these experiments in multiple iPSC lines, which significantly increases the power of our data and substantiates our conclusions. Proteomics as a method of analysis is not without limitations. The sample preparation challenges, reproducibility of separation techniques, challenges associated with detection and quantification of low-abundance proteins, data complexity, and interpretation are all concerns. There also are inter-cell line variabilities between iPSC lines which start at the level of reprogramming and may cause iPSC lines to respond differently to differentiating factors.

## 5. Conclusions

In this study, we demonstrated that DE cells can be efficiently generated with SMs and GFs. The differentiation protocols utilized in these studies are well published and require minimal steps. We show that in the first step of generating DE-derived tissue and organ-specific cells, either protocol can be utilized. However, for hepatic specification, the GF protocol is superior and more efficient.

## Figures and Tables

**Table 1:** qRT-PCR primers.

Sequence Notes	Bases	Sequence
AFP, HOMO_SAPIENS	25	TCT GCA TGA ATT ATA CAT TGA CCA C
	20	AGG AGA TGT GCT GGA TTG TC
FOXA2, HOMO_SAPIENS	19	TGT TCA TGC CGT TCA TCC C
	19	GGA GCG GTG AAG ATG GAA G
SOX17, HOMO_SAPIENS	19	GGC CGG TAC TTG TAG TTG G
	17	AAC GCC GAG TTG AGC AA
HNF4A, HOMO_SAPIENS	21	GAT GTA GTC CTC CAA GCT CAC
	21	GCC ATC ATC TTC TTT GAC CCA
CXCR4, HOMO_SAPIENS	21	GTA CTT GTC CGT CAT GCT TCT
	20	AAA TCT TCC TGC CCA CCA TC
GATA4, HOMO_SAPIENS	18	TTG CTG GAG TTG CTG GAA
	19	GGA AGC CCA AGA ACC TGA A
GAPDH, HOMO_SAPIENS	22	TGT AGT TGA GGT CAA TGA AGG G
	19	ACA TCG CTC AGA CAC CAT G
HHEX, HOMO_SAPIENS	21	CAA ATC TTG CCT CTG ATC ACA
	19	TCA GCG AGA GAC AGG TCA A
CER1, HOMO_SAPIENS	21	GAT GTG TCC ATC TTC ATG CTC
	22	CAC TGA ACT GCA CTG AAC TTT C
SOX9, HOMO_SAPIENS	18	CTT CAG GTC AGC CTT GCC
	18	CAT GAG CGA GGT GCA CTC
FOXA2, HOMO_SAPIENS	19	TGT TCA TGC CGT TCA TCC C
	20	AGC GGG CGA GTT AAA GTA TG

## Data Availability

The datasets used and/or analyzed during the current study are available from the corresponding author on reasonable request. All data generated or analyzed during this study are included in this published article and its Supplementary Information Files.

## Supplementary Materials

The following supporting information can be downloaded at https://www.mdpi.com/article/10.3390/cells14110815/s1. Figure S1. Representative phase-contract micrographs (original magnification 20×) showing morphology of growth factor endoderm cells (GF-ENDs), growth factor hepatoblast cells (GF-HEPBs), small molecule endoderm cells (SM-ENDs), and small molecule hepatoblast cells (SM-HEPBs) following treatment with growth factors and small molecules. Figure S2. Endoderm and hepatoblast TCA cycle metabolomic data. A comparative heatmap of the metabolic profile of 15 iPSCs, SM endoderm, GF endoderm, SM hepatoblast, GF hepatoblast and CTR group replicated to the labeled metabolites (**A**). Volcano plots of the differentially expressed metabolites from the GF endoderm against the SM endoderm group (**B**). The negative x-axis represents downregulated (blue) metabolites in GF endoderm compared to the SM endoderm, and the positive axis represents upregulated (red) metabolites compared to the SM endoderm. Volcano plots of the differentially expressed metabolites from the GF hepatoblasts against the SM hepatoblast (**C**). The negative x-axis represents downregulated (blue) metabolites in the GF hepatoblast compared to the SM hepatoblast, and the positive axis represents upregulated (red) metabolites compared to the SM hepatoblast.

## Declarations

The authors declare that they have not used AI-generated work in this manuscript.

## Abbreviations

The following abbreviations are used in this manuscript:

Human-induced pluripotent stem cells (hiPSCs); hepatocyte-like cells (HLCs); primary human hepatocytes (PHHs); growth factors (GFs); small molecules (SMs); α1-antitrypsin (A1AT); albumin (ALB).
