# Supplementary figures and images for: Efficient Generation of Induced Pluripotent Stem Cell-Derived Definitive Endoderm Cells with Growth Factors and Small Molecules
Source: Cells. 2025 May 30;14(11):815. doi: 10.3390/cells14110815 (PMC12153844; doi:10.3390/cells14110815)

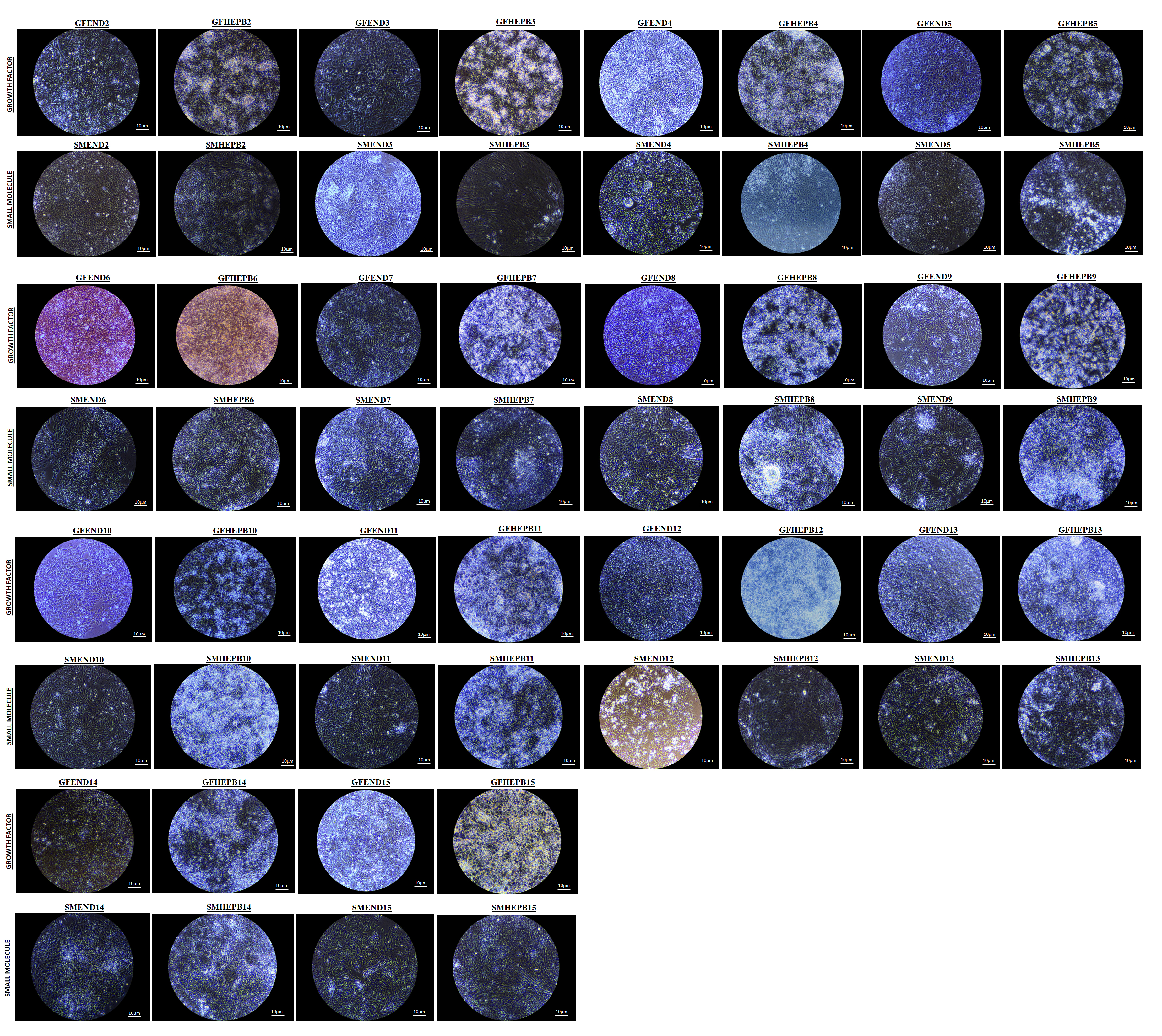

Supplement: Supplementary file 1 [file cells-14-00815-s001.zip › Supplemental Figure S1.jpg]

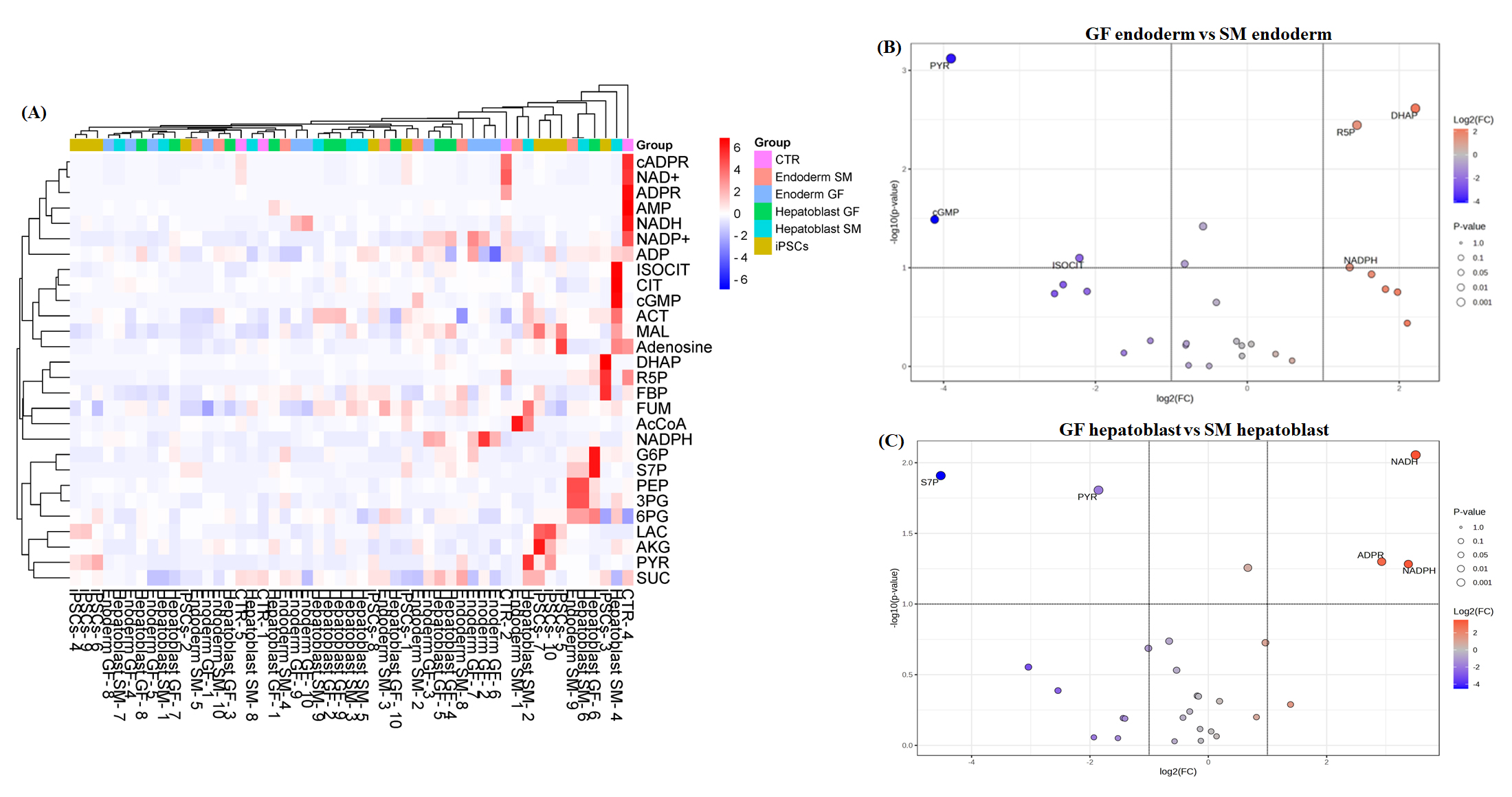

Supplement: Supplementary file 1 [file cells-14-00815-s001.zip › Supplemental Figure S2.jpg]
